# Inpatient care of the elderly in Brazil and India: Assessing social inequalities

**DOI:** 10.1016/j.socscimed.2012.09.015

**Published:** 2012-12

**Authors:** Andrew Amos Channon, Monica Viegas Andrade, Kenya Noronha, Tiziana Leone, T.R. Dilip

**Affiliations:** a Centre for Global Health, Population, Poverty and Policy, University of Southampton, Southampton, UK; b CEDEPLAR, Federal University of Minas Gerais, Brazil; c London School of Economics, London, UK; d Public Health Foundation of India, Delhi, India

**Keywords:** Inpatient, Brazil, India, Inequalities, Health system

## Abstract

The rapidly growing older adult populations in Brazil and India present major challenges for health systems in these countries, especially with regard to the equitable provision of inpatient care. The objective of this study was to contrast inequalities in both the receipt of inpatient care and the length of time that care was received among adults aged over 60 in two large countries with different modes of health service delivery. Using the Brazilian National Household Survey from 2003 and the Indian National Sample Survey Organisation survey from 2004 inequalities by wealth (measured by income in Brazil and consumption in India) were assessed using concentration curves and indices. Inequalities were also examined through the use of zero-truncated negative binomial models, studying differences in receipt of care and length of stay by region, health insurance, education and reported health status. Results indicated that there was no evidence of inequality in Brazil for both receipt and length of stay by income per capita. However, in India there was a pro-rich bias in the receipt of care, although once care was received there was no difference by consumption per capita for the length of stay. In both countries the higher educated and those with health insurance were more likely to receive care, while the higher educated had longer stays in hospital in Brazil. The health system reforms that have been undertaken in Brazil could be credited as a driver for reducing healthcare inequalities amongst the elderly, while the significant differences by wealth in India shows that reform is still needed to ensure the poor have access to inpatient care. Health reforms that move towards a more public funding model of service delivery in India may reduce inequality in elderly inpatient care in the country.

## Highlights

► Increasing numbers of individuals surviving to older ages in Brazil and India means that inpatient care is a growing issue. ► Receipt of inpatient time and length of stay are analysed for adults over 60 years in Brazil in 2003 and India in 2004. ► Both the receipt of care and the length of stay in hospital for elderly in Brazil do not show any inequality by wealth. ► In India the rich are more likely to be admitted to hospital than the poor, but there are no differences for length of stay. ► The higher educated and those with health insurance in both countries are most likely to obtain inpatient care.

## Introduction

Inpatient care is a key aspect of any health system, especially with regards to the treatment of the more vulnerable older adult population. This paper explores the socio-economic inequalities in the probability of receiving inpatient care in the last 12 months in two contrasting settings, India and Brazil, for adults aged over 60 years. Also explored are inequalities in the length of inpatient stay for the same group of adults.

The rapidly increasing older adult population in low and middle income countries provides a challenge for the provision of sufficient healthcare to this group. Elderly populations have a higher prevalence of chronic diseases, spend a larger amount on medicines and demand a greater range of hospital services (Brody, 1988). Furthermore, in many countries treatment in hospital is the main focus of healthcare for the elderly, with a heavy reliance on more expensive acute care services rather than primary or secondary prevention (World Health Organisation, 2002a). Reforming health systems in order to place prevention at the forefront of healthcare for the elderly has been acknowledged to be a major factor in reducing morbidity and expense (World Health Organisation, 2002b).

The aim of this paper is to analyse socio-economic inequalities in inpatient care utilisation of older adults, contrasting two countries with different models of health service delivery, Brazil and India. There are similarities between the countries on many dimensions, with both currently experiencing rapid epidemiological and demographic transitions. Both have similar publicly sponsored social security measures for the elderly (e.g. Bloom, Mahal, Rosenberg, & Sevilla, 2010; de Carvalho Filho, 2008; Midgley, 2012) aside from those related to the healthcare system, with the mainly publicly funded services in Brazil contrasting with a large proportion of services paid by out-of-pocket payments in India (Mahal, Debroy, & Bhandari, 2010; World Health Organisation, 2008).

Demographically there are clear similarities between Brazil and India, although Brazil is at a slightly later stage of the demographic transition with below replacement fertility levels and falling mortality (United Nations Department of Economic and Social Affairs Population Division, 2011). Both Brazil and India are ageing quickly, with 10.3% of Brazilians and 7.6% of Indians over 60 years of age, although this is still lower than the 21.7% of the population over 60 in more developed countries (United Nations Department of Economic and Social Affairs Population Division, 2009). This proportion is forecasted to grow to 18.9% and 12.4% respectively by 2030, totalling over 40 million in Brazil and 184 million in India (United Nations Department of Economic and Social Affairs Population Division, 2009). Income inequality in the two countries is high, with India having a GINI coefficient (measuring income inequality in a population, with 0 indicating complete equality and 100 perfect inequality) of 36.8 while Brazil has a corresponding coefficient of 55.0 (UNDP, 2010).

Brazil and India offer an opportunity to analyse healthcare inequalities in two different contexts. Brazil is a rapidly developing country that still has a high level of poverty and income inequality but has made large advances in re-organising the healthcare system in the last two decades to become a largely government funded system (World Health Organisation, 2008). Conversely India is a developing country with a deeper level of poverty and lower income inequality than Brazil, with a public health system that is not well organised, dominated by out-of-pocket finance and covers only part of the population (Mahal et al., 2010). In Brazil the publicly funded health system, Sistema Unico Saude (SUS) provides universal, integrated and free healthcare services. This sector accounts for the largest proportion of healthcare, while health insurance and family expenditure are also used (Medici, 2002). India's current public health policy focus is to ensure universal coverage for essential healthcare and medicines through the strengthening of public funding (Planning Commission, 2012) and a reduction in private out-of-pocket expenditure. Due to the similarities between the countries, except in regard to the health system organisation, understanding the nature of inequalities in elderly access to inpatient care in Brazil will contribute to the evidence base in relation to health reform in India.

Brazil has made large advances in the last two decades in developing the public healthcare system. The main institutional reform in the Unified Health System relates to primary and preventive care, including the Family Health Program (FHP) which guarantees access to preventive care, especially for low income groups (Paim, Travassos, Almeida, Bahia, & Macinko, 2011). This program has reduced avoidable hospitalisation (Macinko et al., 2010, 2011). In Brazil about 70% of inpatient services are financed by the public system, representing around 50% of the total public health budget (La Forgia & Couttolenc, 2008). The Indian health system is largely reliant on household expenditure with private spending accounting for 78.1% of total health expenditure in 2009 (Ministry of Health and Family Welfare, 2009). Individuals are increasingly using private sector provision due to the higher quality of services available (Sengupta & Nundy, 2005). The cost of private treatment varies widely due to the large range of services available (Baru, 2005), and the private sector is a major provider of inpatient care (Sengupta & Nundy, 2005).

Inequalities in obtaining healthcare among the adult population are well explored in these countries (e.g. Balarajan, Selvaraj, & Subramanian, 2011; Guanais, 2010; Mahal, Yazbeck, Peters, & Ramana, 2001; Wallace & Gutiérrez, 2005), while there is some evidence relating to the inequalities in healthcare services specifically for the elderly population (Lima-Costa, Matos, & Camarano, 2006). The few studies focussing on inpatient care have mainly found little inequality. Blay, Fillenbaum, Andreoli, and Gastal (2008), studying both outpatient and inpatient care among the elderly in Brazil, found that males, those of higher age and those with health insurance more likely to report care, although this study did not investigate differentials by wealth. An earlier study by Almeida, Travassos, Porto, and Labra (2000) highlighted that there were no social inequalities in the use of inpatient services for all the Brazilian population, although regionally there were differential admission rates. Inequality was also seen in a study by Noronha and Andrade (2002), although it was the poor who had a higher probability of being hospitalised with a longer time spent in hospital, potentially due to the poor presenting to the doctor at a later stage of illness and therefore needing a higher level of intensive, inpatient care.

In India inequality varies by state. Mahal et al. (2001) found inequality in favour of the rich for curative care services in all states except for Kerala, where a pro-poor bias was observed. The result for Kerala was confirmed in a recent paper showed increasing hospitalisation within each quintile and a reduction in pro-rich inequality, although the increase in hospitalisation among the poor was through the use of private providers (Dilip, 2010). Further studies have indicated large differences in inpatient care utilisation by state (e.g. Mukherjee & Levesque, 2010; Singh & Ladusingh, 2009).

The contrasting health system organisation in the two countries and the ongoing health reforms provide a context within which inequalities can be assessed for both inpatient care and length of stay in hospital. The paper highlights current issues relating to inequality in the two countries on these dimensions, while enabling Indian health system reformers to study the Brazilian context and ensuring lessons learnt are applied to their own context.

## Data

The Brazilian National Household Survey 2003 (PNAD) and the Indian National Sample Survey Organisation (NSSO) data from 2004 are used. These two datasets are comparable on many dimensions, with information collected about health of selected individuals by state. PNAD is the main household survey in Brazil and information is collected about health and morbidity every five years. In India, the 60th NSSO had a focus on morbidity and healthcare. Both PNAD and NSSO record the utilisation of healthcare for both inpatient and outpatient care, with inpatient episodes recorded in detail. However, detailed questions on this are asked in the NSSO only to those aged over 60 years, and hence both datasets were restricted to this age range. This study is secondary analysis of data so ethical approval was not required. Ethical approval was obtained for the original surveys.

## Variables used in the study

Wealth can be measured in many different ways, which have been much debated elsewhere (e.g. O'Donnell, Van Doorslaer, Wagstaff, & Lindelow, 2008). In PNAD both assets and income are recorded, while for NSSO limited information about household assets are collected alongside household consumption. Income in Brazil and consumption in India per capita were therefore used. For India consumption in the household is based on a 30-day recall period and consists of all purchases in the household, the value of goods consumed from home stock, value of receipts in exchange for goods and services, the value of gifts and loans and the value of any other free items consumed. For less frequent purchases, such as clothing and education, the total spent in the last year was recorded and divided by 12 to get an approximate monthly value. The total of all consumption items is calculated and divided by the household size. Income and consumption per capita are not directly comparable as they are measuring different components of wealth. However, previous research has demonstrated that when used as relative measures of wealth that there is comparability (O'Donnell et al., 2007).

Two outcome variables are analysed:

1) The receipt of inpatient care in the year prior to the survey;
2) Length of stay in hospital if inpatient care was obtained.

There are two stages related to the process of receiving inpatient care. The first stage relates to the probability of receiving inpatient care. This probability may depend on both individual and providers' characteristics. Individuals who are less educated, living far from healthcare establishments and who have a low expectancy of receiving healthcare have a lower probability of seeking health services (e.g. Mahal et al., 2001; Roy & Chaudhuri, 2008). The primary service providers also influence hospitalisation as they are usually responsible for the decision to refer the patient to hospital. The second stage of receiving inpatient care is related to the decision about how long the patient should stay in hospital. This decision depends on the severity of the disease, the patients' characteristics and again on the incentives for the providers. Inequality can be present at both of these stages.

A number of variables was used as controls in the model, including region, place of residence, education, gender, age, self-reported health status and receipt of health insurance. The descriptions of these controls are given in Appendix A. Age was treated as a continuous measure, although a categorised variable was also tested. However, no large differences were seen by age group and the predictive power of the models was superior when treated as a continuous variable. Individuals were identified as having had an inpatient care episode in the last 365 days via a section of the questionnaire which asked about the details of each inpatient episode in the household including the length of each stay. Survey design weights were used in all analyses. The sample size after restricting the datasets to adults over 60 years old was 35,114 for Brazil and 34,745 for India.

## Methodology

To evaluate the relationship between socio-economic status and inpatient care two methods were used: 1) concentration indices and curves and 2) hurdle negative binomial models.

The Concentration Index (CI) quantifies wealth-related inequality in a health related variable (O'Donnell et al., 2008), measuring the relationship between the cumulative proportion of population ranked by socio-economic status against the cumulative proportion of health related outcome variable. The CI ranges from −1 to +1, with a CI equal to zero indicating an absence of social inequality and one equal to −1 or +1 indicating that the health related outcome variable is totally concentrated in the poorest or wealthiest individuals respectively. OLS regression was used to estimate the CI (equation (1))

(1) $$2\sigma_{r}^{2}\left(\frac{h_{i}}{\mu }\right)=\alpha +\beta r_{i}+\sum_{j}\delta_{j}x_{ij}+\upsilon_{i}$$

where *r*_*i*_ is the fractional rank of the individual *i* in the socio-economic distribution, *σ*_*r*_^2^ is the fractional rank variance, *h*_*i*_ is the inpatient care indicator for individual *i* and *μ* is its mean. Control variables are included in the *x*-vector and *v*_*i*_ is the error term. *β* is the estimated concentration index.

The graphical representation of the CI is the concentration curve (CC). The diagonal represents perfect equality in the distribution (a CI equals to zero). For CC below (above) the diagonal, the CI is positive (negative). The CC can also cross the diagonal and in these cases, the calculation of the CI does not reflect the total magnitude of social inequality.

The hurdle negative binomial model is the econometric model generally used to estimate social inequality in the healthcare utilisation (Mullahy, 1986). This model takes into account the main particularities associated to the decision-making process underlying the demand for healthcare. First, the number of days in hospital only takes non-negative integer values. In addition, the distribution of these events is similar to the Poisson distribution, indicating that the probability of an occurrence of an event (i.e. using healthcare services) reduces when its frequency (i.e. number of days in the hospital) increases. This characteristic requires count data models such as the negative binomial model to be estimated. Secondly, the use of healthcare services may have a skewed distribution with a large amount of zeros. Therefore, the estimation of zero-truncated model is appropriate.

The two-stage estimation is crucial to identify which factors affect the decision of the patient to visit a physician and the decision of the doctor when determining the amount of treatment for each patient. Utilisation may be considered as two different stochastic processes as the physician who decides whether an individual should be hospitalised is usually different from the one who decides the length of stay. The hurdle negative binomial model estimates the hospitalisation and duration decisions separately.

Let *y*_*i*_ be the number of days that individual *i* stayed in the hospital, with *y*_*i*_ ≥ 0, and define *d*_*i*_ as a binary variable equal to 1 if individual was admitted to the hospital. The likelihood function for the hurdle negative binomial model $L_{\text{BN}}^{H}$ may be specified as follows:

(2) $$L_{\text{BN}}^{H}=\prod_{i\in \Omega }\text{pr}{\left\{y_{i}=0|x_{i}^{\prime }\beta_{1},\alpha_{1}\right\}}^{1-d_{i}}{\left(1-\text{pr}\left\{y_{i}=0|x_{i}^{\prime }\beta_{1},\alpha_{1}\right\}\right)}^{d_{i}}\times \prod_{i\in \Omega_{1}}\frac{\text{pr}\left\{y_{i}|x_{i}^{\prime }\beta_{2},\alpha_{2}\right\}}{\text{pr}\left\{y_{i}\geq 1|x_{i}^{\prime }\beta_{2},\alpha_{2}\right\}}$$

where: *i*, 1, 2, 3, …, individuals; *α*_*s*_, overdispersion parameter of data in each stage, with *s* = 1, 2; *Ω* = whole sample; *Ω*_1_, subsample that considers only individuals admitted to the hospital.

The first part of the likelihood function is estimated considering the whole sample *Ω* and it represents the binary decision process defining whether individual is admitted to the hospital. In this stage logistic regression models are used to estimate the vector of parameters (*β*_1_, *α*_1_). The second part of the likelihood function considers only the individuals who were admitted to hospital (*Ω*_1_). A truncated-at-zero negative binomial model is used to estimate the expected number of days in the hospital and the vector of parameters (*β*_2_, *α*_2_). The coefficients of this model can be interpreted as the log of the expected number of days spent in hospital, with negative values indicating fewer days while positive values indicate a longer time in hospital.

## Descriptive results

Appendix B shows descriptive statistics for Brazil and India by quintiles of income and consumption respectively alongside place of residence and health insurance. These indicate only slight differences by quintile for hospitalisation and length of stay in hospital within Brazil, although the percentage of those who are insured and the average years of schooling increase markedly with income quintiles. India, in contrast to Brazil, displays some differences by consumption quintiles in the probability of hospitalisation, with only 5% of the poorest reporting a hospital episode, compared to 7.5% in the richest group.

Differentials are much more pronounced in India between rural and urban areas. The percentage of older adults hospitalised is 5.3% in rural areas while it is 8.6% in urban areas. For Brazil health indicators are similar irrespective of the place of residence. The comparison of individuals with and without health insurance reveals some differences in Brazil, with insured elderly individuals reporting higher hospitalisation (14.3% against 12.0%). For India the insured population is very small but there are great disparities between the insured and non-insured population.

## Receipt of inpatient care

**Fig. 1:**
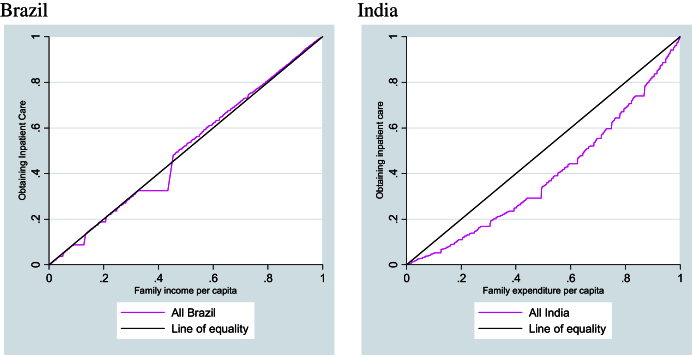
Concentration curves for obtaining inpatient care for Brazil and India.

Fig. 1 shows the concentration curves for obtaining inpatient care for both Brazil and India. The curve for Brazil indicates an absence of income inequality. In India, socio-economic inequalities are clearly observed, with hospitalisation favouring richer groups.

The concentration index indicates significant pro-poor inequality in inpatient care in Brazil before controls are included and also after controlling for sex and age, as evidenced by the significant negative result obtained (Table 1). However, since the concentration curve crosses the diagonal (as seen in Fig. 1), this inequality is not verified along the total range of the income distribution. Therefore, it is not possible to say that there is socio-economic inequality in receiving inpatient care favouring poor individuals before other variables are taken into account. After all the control variables are included there is no inequality indicated at all – the index shows almost complete equality with a value of almost zero.

For India, in contrast to Brazil, there is significant socio-economic inequality favouring the richer groups, shown by the significant positive index score. The value of the concentration index decreases with the inclusion of control variables, with the greatest reductions observed when education and region of residence are included in the calculations. Even after including all the control variables the concentration index is still significant indicating pro-rich bias in hospitalisation (Table 1).

The results of the logistic model for the receipt of inpatient care are shown in Table 2 for Brazil and Table 3 for India. Eight models for each country were estimated, with the first only including income/consumption, while the remaining models included a selection of controls. The final model includes all control variables.

The results for Brazil indicate that there are minimal inequalities in the receipt of inpatient care by income once age and sex have been controlled for. Model 2 shows that those in the second quintile are actually more likely to have been admitted to hospital than the richest quintile. However, after self-reported health is controlled for (Model 3) there is a pro-rich bias observed, with the richest individuals more likely to be hospitalised than a poorer person for the same level of health. The inclusion of schooling, household size, region and urban/rural area does not alter the relationship (Models 4–7). However, after controlling for health insurance (Model 8) there is no longer significant socio-economic inequality. This indicates that health insurance is an important mechanism that generates socio-economic inequality in the receipt of inpatient care in Brazil among elderly people. Health insurance has a strong relationship with care. As shown in Appendix A the rich are most likely to have health insurance. Hence there is socio-economic inequality in care driven by health insurance differentials.

In the final model (Model 8) for Brazil there are wide differentials by education, with the odds of less educated individuals having an inpatient episode much lower than those who attended high school or more. This relationship may be seen due to lower reporting amongst the lower educated, or it may be related to a lower need for inpatient care for those with worse education. The logistic model also indicates that females obtain less care than males, those at older ages are more likely to obtain care than at younger ages and self-rated health has a strong relationship with inpatient care. Self-rated health needs to be treated with caution however, as this is self-rated at the time of the survey, thus after any inpatient care episode. Hence a person may describe their health as poor only *after* the inpatient visit.

In comparison, there are clear inequalities by consumption in obtaining inpatient care in India, indicated by the significant odds ratios in Table 3. After controlling for other important explanatory variables, there is no significant difference in the receipt of inpatient care between the richest two quintiles (Model 8). However, the odds of inpatient care for the poorest three quintiles are significantly lower compared with the richest group. There are also inequalities observed by education, with the illiterate group much less likely to have received inpatient care than the most educated group. The remaining control variables show relationships in the expected direction. All regions had significantly reduced odds of inpatient care compared to the Southern region.

## Length of stay

**Fig. 2:**
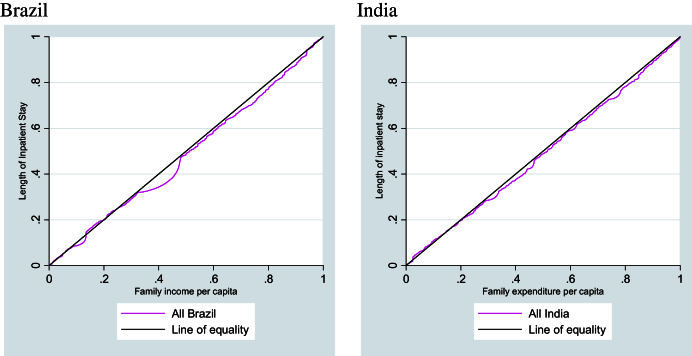
Concentration curves for the length of inpatient stay for Brazil and India.

Fig. 2 displays the concentration curves for the length of time that hospital care was received for both Brazil and India.

It is clear from the concentration curves that there is no inequality in either country. The concentration indices for both Brazil and India are not significant before controls are taken into account (Table 4). However, in India once urban/rural dwelling is accounted for, the index is seen to be significant. The index is positive but of low magnitude, indicating that the richer groups are more likely to have longer inpatient stays.

Table 5 shows the results of the zero-truncated negative binomial model for Brazil, while Table 6 shows the same for India.

For the majority of models for Brazil it is clear that income does not show a relationship with the length of inpatient stay. It is also noticeable that the presence of health insurance does not relate significantly to the hospitalisation duration (Model 8). However, in all models where it has been included (Models 4–8) education does indicate a relationship, with those without any schooling spending a shorter time in hospital compared with those with High School or greater education. Females spend less time in hospital than males.

For India there are differences by consumption, with individuals in the third and fourth richest quintiles having a significantly shorter length of stay than the richest quintile (shown in all models). Differences between the other quintiles are not significant. Interestingly health insurance is significant (Model 8), with those with insurance spending a shorter length of time in hospital than those without. Although those with health insurance are also most likely to be in the richest quintile, who have the longest hospital stays, after controlling the model for wealth the reduced length of stay for those with insurance may indicate better quality of care for insurance holders compared to those without insurance. Alternatively it may be that those with insurance are admitted with more minor ailments, therefore needing less time as an inpatient. Self-reported health is again related to length of stay, in the expected direction in the final model.

## Discussion

This study has utilised two nationally representative datasets in two of the largest and fastest growing economies in the world to contrast patterns of inequalities with respect to two measures of inpatient care in older adults. The first measure relates to the receipt of inpatient care, while the second refers to the length of time that inpatient care was received, conditional on receipt. Inequalities were measured on a range of dimensions, with the key economic aspects of income and consumption studied.

The results indicated clear differences in economic inequality between Brazil and India with respect to the receipt of inpatient care. In Brazil there was no evidence observed for economic inequality, while in India those who were classified as rich were more likely to be admitted to hospital. Inequality was seen in both countries by sex, region, education and health insurance, with those who have insurance more likely to have received care. This similarity between the countries on other dimensions of inequality further highlights the difference between them with respect to inequalities by wealth. In Brazil, once self-reported health status was controlled for a richer individual was more likely to have received care than a poorer individual, indicating that receipt of care may be linked to health – wealthier individuals obtain care for less serious illnesses or for preventative care, although it is not possible to verify this using these datasets. There was no income or consumption inequality in either country in regard to the length of stay in hospital – irrespective of the wealth of the older adult the reported stay was, on average, the same. This indicates that once care is obtained there is no discrimination in the amount of care received by wealth. Yet there is a relationship with education in Brazil, with those with less education having a shorter time in hospital. Evidence does indicate that educational level is related to wealth in Brazil (e.g. Murakami & Blom, 2008; Torche & Costa-Ribeiro, 2012), so this inequality may simply be reflecting wealth differentials.

The absence of inequality by wealth in Brazil is consistent with other studies in higher income countries (Allin, Masseria, & Mossialos, 2010; Goddard & Smith, 2001), indicating the progress made by the health system in the country in ensuring equitable access. There has been a move to a preventative system of healthcare using primary health services which has reduced avoidable hospitalisation (Macinko et al., 2010) and could be hypothesised as being the driver for reducing inequalities in obtaining inpatient care. However, the richest individuals with insurance are seen to be more likely to obtain inpatient care, indicating that they can attend both the public and private sectors, while the poorest rely on the public sector only.

The lack of inequality in Brazil highlights a potential pathway for the Indian health system reforms. A reduction in the private sector and a strengthening of public provision are an aim of the Indian reforms (Planning Commission, 2012), similar to the process followed by Brazil. The higher inequity observed in India can be partly attributed to the financial burden of care. Inpatient care provision in the private sector is increasing throughout the country (Gangolli, Duggal, & Abhay, 2005), where the majority of the financial burden falls on individuals and households (Balarajan et al., 2011). For older adults, who rely on family, friends and other social structures for support may not have the funds required for care. The cross-sectional nature of the data did not allow the examination of whether poverty is a result of catastrophic healthcare expenses or whether low income was the key barrier to healthcare access.

This study only indicates whether inpatient care was received or not and does not investigate the type of care that was obtained due to a lack of this available information in the datasets. It may be hypothesised that the richer adults receive more preventative care in general and are more likely to receive elective inpatient care. In contrast, poorer individuals are more likely to receive emergency inpatient care (Allin et al., 2010; Goddard & Smith, 2001). This issue is especially important for elderly individuals as preventative care is essential to detect early onset of diseases and to postpone the adverse consequences associated to them such as disability. Furthermore, in both countries these results do not shed light on inequalities related to access to primary or outpatient care services.

## Limitations

This paper comes with a series of limitations, mainly linked to the data used. First, the datasets utilised were collected for different reasons in Brazil and India. A comparative paper would ideally use the same variables in all countries, but this was not possible in this study. This is particularly the case for the measure of wealth. However, the microdata exploited for comparative research is a major strength of this study and indicates that such a comparison can be made with imperfectly matching datasets. The quality of the survey instruments has been highlighted in other studies (Gumber & Berman, 1997; Murray & Chen, 1992).

Related to this, the cultural context in both countries was not taken into account during the modelling due to a lack of comparable data. Understanding the relationship between caste and access to healthcare in India would highlight inequalities in this regard, but there is no comparable variable in the Brazilian dataset. The use of traditional medicine may also differ between the countries and especially amongst the older cohorts. If traditional methods are preferred by some groups in the analysis then inequalities may be observed in access to inpatient care where in fact none exist.

Second, issues of endogeneity are not addressed in this paper. The presence of endogeneity biases coefficient's estimates. In this paper two sources of endogeneity are possibly present. The first relates to the receipt of inpatient care and socio-economic status (SES) and whether the receipt of care actually will influence the recorded SES. However, this paper only analyses the relationship between variation in SES and inpatient care, while the causal interpretation between both variables is beyond the scope of this study. The second source relates to inpatient care and self-reported health status, as individual health status was evaluated after receiving inpatient care. Therefore inpatient care could have affected the present health status. It can be assumed that present health status is correlated to past health status and hence this endogeneity is not of a large degree. Longitudinal databases or instrumental variable estimations would be solutions to address these issues. Unfortunately the datasets available in Brazil and India do not allow this to be taken into account.

The data available also precluded investigations into the quality and type of care received, both of which are critical when considering inequalities in healthcare. More extensive international collaborations in data collection, similar to the Living Standards Measurement Surveys (LSMS) and the Survey of Health, Ageing and Retirement in Europe (SHARE) are needed to obtain comparative information on healthcare access and consumption. The value of comparative analyses is fundamental in order that lessons can be learnt from countries that have undergone substantial health system reforms, such as Brazil, or that are undergoing similar patterns of population ageing.

## Conclusions

This study is particularly relevant in the light of the Indian government's aims to strengthen the public provision within the country. The success of Brazil's health reforms in reducing inequalities in elderly inpatient care indicates a potential pathway that could be followed. However, there is some indication that inequalities do remain in Brazil, with differentials by education potentially indicating wealth-related inequality. The high levels of inequality in India highlight the difficulties faced by the elderly poor under the current system in obtaining the care that they require.

The analysis of health inequalities of older adults in low and middle income countries is very opportune at a time of health systems reforms and resource allocation reshuffling. Too often older adults are grouped with the adult population and as a result some of the key issues that are faced by this group are neglected. The allocation of resources within households in settings where fertility may still be above replacement, especially amongst the poor, could potentially lead to an overall lack of funds for the older members of the family as attention is still focused on the young. Furthermore the protective effect of families and relationships across wealth groups needs to be understood (Grundy & Sloggett, 2003). If inpatient care is not received when needed then this could eventually result in long term care issues which would be more expensive for both the household and the health system.

This study has highlighted that inequalities persist in both countries with respect to older adults receiving inpatient care, although a lack of differentials by wealth in Brazil indicates the progress made by the health system in encouraging health for all. In India wealth is still related to obtaining inpatient care, signifying the effect of out-of-pocket costs of care. Both countries suffer from inequalities by region and education – revealing that there are persistent differences between groups that should be targeted. The care needs of older adults are specific to this group and the health system in both Brazil and India should respond to the rapidly increasing numbers of these groups to provide the level of care needed, irrespective of location, education or, in the case of India, wealth.

## Figures and Tables

**Table 1:** Concentration indices for obtaining inpatient care for Brazil and India.

	Brazil	India
No controls	−0.018*	0.209*
+Sex/Age	−0.018*	0.203*
+Health Status	0.039*	0.228*
+Education	0.035*	0.188*
+Household Size	0.035*	0.196*
+Region	0.032*	0.167*
+Rural	0.031*	0.160*
+Health Insurance	−0.002	0.159*

*p < 0.05.

**Table 2:** Odds ratios for receipt of inpatient care in the last year in Brazil.

Variables		(1) No control	(2) Sex/Age	(3) Health status	(4) Schooling	(5) Household size	(6) Region	(7) Rural	(8) Health insurance
Income quintile	1st	1.06NS	1.08NS	0.79***	0.81***	0.81***	0.83***	0.83***	1.01NS
	2nd	1.17***	1.11*	0.83***	0.85***	0.85***	0.85**	0.86**	1.03NS
	3rd	1.04NS	1.04NS	0.86**	0.88*	0.88*	0.88*	0.88*	1.02NS
	4th	0.97NS	0.97NS	0.85***	0.87**	0.87**	0.87**	0.87**	0.96NS
Sex	Female		0.91***	0.89***	0.89***	0.89***	0.90***	0.90***	0.88***
Age			1.03***	1.03***	1.03***	1.03***	1.03***	1.03***	1.03***
Self-Reported Health	Excellent			0.12***	0.12***	0.12***	0.12***	0.12***	0.12***
	Good/fair			0.31***	0.31***	0.31***	0.31***	0.31***	0.31***
Education	Illiterate				0.89NS	0.89NS	0.89NS	0.90NS	1.03NS
	Primary				0.90NS	0.90NS	0.88*	0.88*	0.97NS
	Middle				0.76***	0.76***	0.73***	0.73***	0.78***
Household size						1.00NS	1.00NS	1.00NS	1.00NS
Region	Southeast						0.73***	0.72***	0.74**
	North						0.66***	0.66***	0.68***
	Northeast						0.65***	0.65***	0.66***
	South						0.98NS	0.99NS	1.01NS
Type of place of residence	Rural							1.04NS	0.98NS
Health Insurance	Yes								1.53***

***p < 0.01, **p < 0.05, *p < 0.1. NS = Not Significant.

**Table 3:** Odds ratios for receipt of inpatient care in the last year in India.

Variables		(1) No control	(2) Sex/Age	(3) Health status	(4) Schooling	(5) Household size	(6) Region	(7) Rural	(8) Health insurance
Income quintile	1st	0.67***	0.67***	0.62***	0.78***	0.78***	0.68***	0.63***	0.63***
	2nd	0.75***	0.76***	0.71***	0.84**	0.84**	0.76***	0.72***	0.72***
	3rd	0.75***	0.76***	0.73***	0.83**	0.83**	0.76***	0.72***	0.73***
	4th	0.86**	0.87*	0.85**	0.94NS	0.94NS	0.91NS	0.88NS	0.88NS
Sex	Female		0.80***	0.75***	0.94NS	0.94NS	0.88**	0.84***	0.84***
Age			1.03***	1.01***	1.01***	1.01***	1.01***	1.01***	1.01***
Self-Reported Health	Excellent			0.16***	0.14***	0.14***	0.13***	0.13***	0.13***
	Good/fair			0.42***	0.40***	0.40***	0.37***	0.36***	0.36***
Education	Illiterate				0.47***	0.47***	0.52***	0.66***	0.68***
	Literate below primary				0.82*	0.82*	0.85NS	1.01NS	1.03NS
	Primary complete				1.05NS	1.05NS	0.98NS	1.14NS	1.17NS
	Secondary school				0.85NS	0.85NS	0.79*	0.84NS	0.85NS
Household size						1.00NS	1.03***	1.03***	1.03***
Region	North						0.57***	0.56***	0.56***
	Central						0.35***	0.35***	0.35***
	East						0.35***	0.35***	0.35***
	North East						0.36***	0.37***	0.37***
	West						0.84**	0.81***	0.80***
Type of place of residence	Rural							0.72***	0.72***
Health Insurance	Yes								1.59**

***p < 0.01, **p < 0.05, *p < 0.01. NS = Not Significant.

**Table 4:** Concentration indices for length of inpatient stay for Brazil and India.

	Brazil	India
No control	0.021	0.015
+Sex/Age	0.022	0.016
+Health Status	0.064*	0.024
+Education	0.020	0.023
+Household Size	0.021	0.027
+Region	0.008	0.029
+Rural	0.007	0.037*
+Health Insurance	0.016	0.038*

*p < 0.05.

**Table 5:** Coefficients for zero-truncated negative binomial Model for length of inpatient stay in Brazil.

Variables		(1) No control	(2) Sex/Age	(3) Health status	(4) Schooling	(5) Household size	(6) Region	(7) Rural	(8) Health insurance
Income quintile	1st	−0.09NS	−0.09NS	−0.24*	−0.01NS	0.00NS	0.09NS	0.08NS	0.05NS
	2nd	−0.21NS	−0.21*	−0.32***	−0.11NS	−0.11NS	−0.05NS	−0.05NS	−0.08NS
	3rd	−0.21NS	−0.21NS	−0.25**	−0.07NS	−0.06NS	−0.03NS	−0.04NS	−0.06NS
	4th	−0.20NS	−0.18NS	−0.18NS	−0.05NS	−0.05NS	−0.00NS	−0.01NS	−0.02NS
Sex	Female		−0.18**	−0.18**	−0.16**	−0.16**	−0.15**	−0.15**	−0.14**
Age			0.01**	0.01*	0.01**	0.01**	0.01**	0.01**	0.01**
Self-Reported Health	Excellent			−1.38***	−1.42***	−1.42***	−1.44***	−1.45***	−1.45***
	Good/fair			−0.65***	−0.66***	−0.66***	−0.67***	−0.67***	−0.67***
Education	Illiterate				−0.46***	−0.46***	−0.43***	−0.40**	−0.42**
	Primary				−0.23*	−0.23*	−0.26*	−0.24*	−0.26*
	Middle				−0.16NS	−0.16NS	−0.13NS	−0.13NS	−0.13NS
Household size						−0.01NS	0.00NS	0.00NS	0.00NS
Region	Southeast						−0.03NS	−0.04NS	−0.05NS
	North						0.01NS	0.04NS	0.03NS
	Northeast						0.36NS	0.36NS	0.36*
	South						0.13NS	0.16NS	0.16NS
Type of place of residence	Rural							−0.21**	−0.22**
Health Insurance	Yes								−0.08NS
Constant		1.69***	0.93NS	1.75***	1.67***	1.71***	1.49***	1.53***	1.57***

***p < 0.01, **p < 0.05, *p < 0.01. NS = Not Significant.

**Table 6:** Coefficients for zero-truncated negative binomial Model for length of inpatient stay in India.

Variables		(1) No control	(2) Sex/Age	(3) Health status	(4) Schooling	(5) Household size	(6) Region	(7) Rural	(8) Health insurance
Income quintile	1st	−0.09NS	−0.08NS	−0.08NS	−0.08NS	−0.09NS	−0.09NS	−0.08NS	−0.09NS
	2nd	−0.08NS	−0.08NS	−0.08NS	−0.08NS	−0.09NS	−0.11NS	−0.09NS	−0.10NS
	3rd	−0.14*	−0.13*	−0.13*	−0.13*	−0.14*	−0.16**	−0.16**	−0.16**
	4th	−0.21**	−0.21**	−0.22***	−0.21***	−0.23***	−0.23***	−0.23***	−0.23***
Sex	Female		−0.09*	−0.11**	−0.11**	−0.11**	−0.10*	−0.09*	−0.09*
Age			0.00NS	−0.01NS	−0.01NS	−0.00NS	−0.01NS	−0.00NS	−0.00NS
Self-Reported Health	Excellent			−0.48***	−0.48***	−0.49***	−0.50***	−0.49***	−0.49***
	Good/fair			−0.36***	−0.36***	−0.36***	−0.34***	−0.34***	−0.34***
Education	Illiterate				0.01NS	0.01NS	−0.01NS	−0.03NS	−0.05NS
	Literate below primary				−0.02NS	−0.03NS	−0.06NS	−0.08NS	−0.10NS
	Primary complete				0.04NS	0.03NS	−0.00NS	−0.02NS	−0.04NS
	Secondary school				0.09NS	0.08NS	0.06NS	0.05NS	0.05NS
Household size						0.01NS	0.01NS	0.01NS	0.01NS
Region	North						−0.01NS	0.02NS	0.01NS
	Central						−0.18*	−0.17*	−0.17*
	East						−0.03NS	−0.03NS	−0.03NS
	North East						0.02NS	0.02NS	0.03NS
	West						−0.21***	−0.21***	−0.21***
Type of place of residence	Rural							0.03NS	0.03NS
Health Insurance	Yes								−0.48**
Constant		2.35***	2.46***	2.95***	2.93***	2.87***	2.96***	2.94***	2.97***

***p < 0.01, **p < 0.05, *p < 0.01. NS = Not Significant.

## Footnotes

Appendix A Supplementary data related to this article can be found at http://dx.doi.org/10.1016/j.socscimed.2012.09.015.
