# Supplementary material for: Inpatient care of the elderly in Brazil and India: Assessing social inequalities
Source: Soc Sci Med. 2012 Dec;75(12):2394–402. doi: 10.1016/j.socscimed.2012.09.015 (PMC3657183; doi:10.1016/j.socscimed.2012.09.015)
Supplement: Supplementary file 1 [file mmc1.docx]

**Appendix A: Description of Control Variables used in Brazil and India**

| Variable | Brazil | India |
| --- | --- | --- |
| Sex | Sex was coded dichotomously, with male as the reference category. | |
| Age | Age was used a continuous variable in both countries | |
| Self-Reported Health Status | The original question had five categories of response, which were recoded into 3 groups to mirror those in India. The categories are 1. Excellent; 2. Good and Fair 3. Bad and Very Bad Health. The final category group is used as the reference category | The categories in the questionnaire were 1. Excellent/Very Good 2. Good/Fair 3. Poor. These were left as originally coded, with Poor as the reference group |
| Education | The original education variable measured years of schooling. It was categorise into 4 groups. 1. Illiterate (zero years of schooling) 2. More than zero years of schooling until primary education (1 to 5 years of schooling) 3. More than primary education until secondary education (6 to 9 years of schooling) 4. At least one year of undergraduate education (10 to 16 years of schooling). The final category is used as reference category. | The original education variable was recoded into five groups to capture the diversity of educational outcomes in India. These categories were 1. Not literate 2. No schooling or below primary school but literate 3. Primary or Middle school achieved 4. Secondary school achieved 5. Above secondary school achieved. The final category was used as the reference category. |
| Region | The 26 states were grouped into 5 regions, following established groupings (Southeast, North, Northeast and South), with the Middle West used as the reference region. | Indian states were grouped into 6 regions following established groupings, with the South used as the reference group. |
| Type of place of residence | The urban/rural classification as defined in the survey was used in both countries. Urban was used as the reference category. | |
| Receipt of Health Insurance | This is a dichotomous variable which equals 1 if health insurance is held, which is the reference category | |
| Inpatient Care | Individuals were identified as having had an inpatient care episode in the last 365 days via a section of the questionnaire which asked about the details of each inpatient episode in the household. | |
