# Supplementary material for: Inpatient care of the elderly in Brazil and India: Assessing social inequalities
Source: Soc Sci Med. 2012 Dec;75(12):2394–402. doi: 10.1016/j.socscimed.2012.09.015 (PMC3657183; doi:10.1016/j.socscimed.2012.09.015)
Supplement: Supplementary file 2 [file mmc2.docx]

**Appendix B: Descriptive results for Brazil and India by socioeconomic quintile**

|  | **Socioeconomic quintile** | | | | |  | **Place of Residence** | |  | **Health Insurance** | |  | **Overall** |
| --- | --- | --- | --- | --- | --- | --- | --- | --- | --- | --- | --- | --- | --- |
|  | Poorest | Poorer | Middle | Richer | Richest |  | Rural | Urban |  | Insured | Not Insured |  |  |
|  | **Brazil** | | | | | | | | | | | | |
| Average age | 68.7 | 71.1 | 69.4 | 69.4 | 69.5 |  | 69.6 | 69.8 |  | 69.7 | 69.7 |  | 69.7 |
| Average years of schooling | 1.5 | 1.7 | 2.5 | 3.7 | 7.6 |  | 1.4 | 3.9 |  | 6.4 | 2.3 |  | 3.5 |
| Average HH income (per capita) | - | - | - | - | - |  | 305.8 | 589.1 |  | 1100.8 | 315.5 |  | 541.9 |
| Average days in hospital (for those hospitalised) | 8.5 | 7.8 | 7.8 | 7.9 | 9.2 |  | 0.9 | 1.1 |  | 8.3 | 8.2 |  | 1.0 |
| % Female | 52.7 | 57.0 | 55.3 | 57.6 | 56.4 |  | 49.2 | 57.3 |  | 59.2 | 54.6 |  | 55.9 |
| % Health insurance | 5.8 | 11.0 | 21.2 | 35.9 | 70.4 |  | 8.8 | 33.5 |  | - | - |  | 29.4 |
| % Hospitalised | 12.8 | 13.9 | 12.6 | 11.8 | 12.2 |  | 13.0 | 12.7 |  | 14.3 | 12.1 |  | 12.7 |
| Sample Size | 6,352 | 9,113 | 4,446 | 7,098 | 7,047 |  | 5,813 | 29,301 |  | 10,322 | 24790 |  | 35,114 |
|  | **India** | | | | | | | | | | | | |
| Average age | 67.5 | 67.4 | 67.2 | 67.4 | 67.6 |  | 67.3 | 68.0 |  | 66.6 | 67.4 |  | 67.4 |
| Average HH consumption (per capita) | - | - | - | - | - |  | 538.8 | 1085.0 |  | 2145.0 | 664.6 |  | 671.4 |
| Average days in hospital (for those hospitalised) | 11.2 | 11.3 | 10.7 | 10.1 | 12.1 |  | 11.3 | 10.7 |  | 7.8 | 11.1 |  | 11.1 |
| % Female | 52.2 | 51.6 | 49.0 | 48.8 | 48.6 |  | 49.7 | 51.1 |  | 41.4 | 50.0 |  | 50.0 |
| % Health insurance | 0.2 | 0.1 | 0.3 | 0.4 | 1.3 |  | 0.1 | 1.6 |  | - | - |  | 0.5 |
| % Hospitalised | 5.1 | 5.8 | 5.7 | 6.5 | 7.5 |  | 5.4 | 8.6 |  | 13.8 | 6.1 |  | 6.2 |
| Sample Size | 6,869 | 6,666 | 6,974 | 7,027 | 7,209 |  | 26,308 | 8,437 |  | 160 | 34585 |  | 34,745 |

Source: PNAD/2003 and NSSO-2003

Note: Quintiles are measured in family income per capita in Brazil and household consumption per capita for India.
